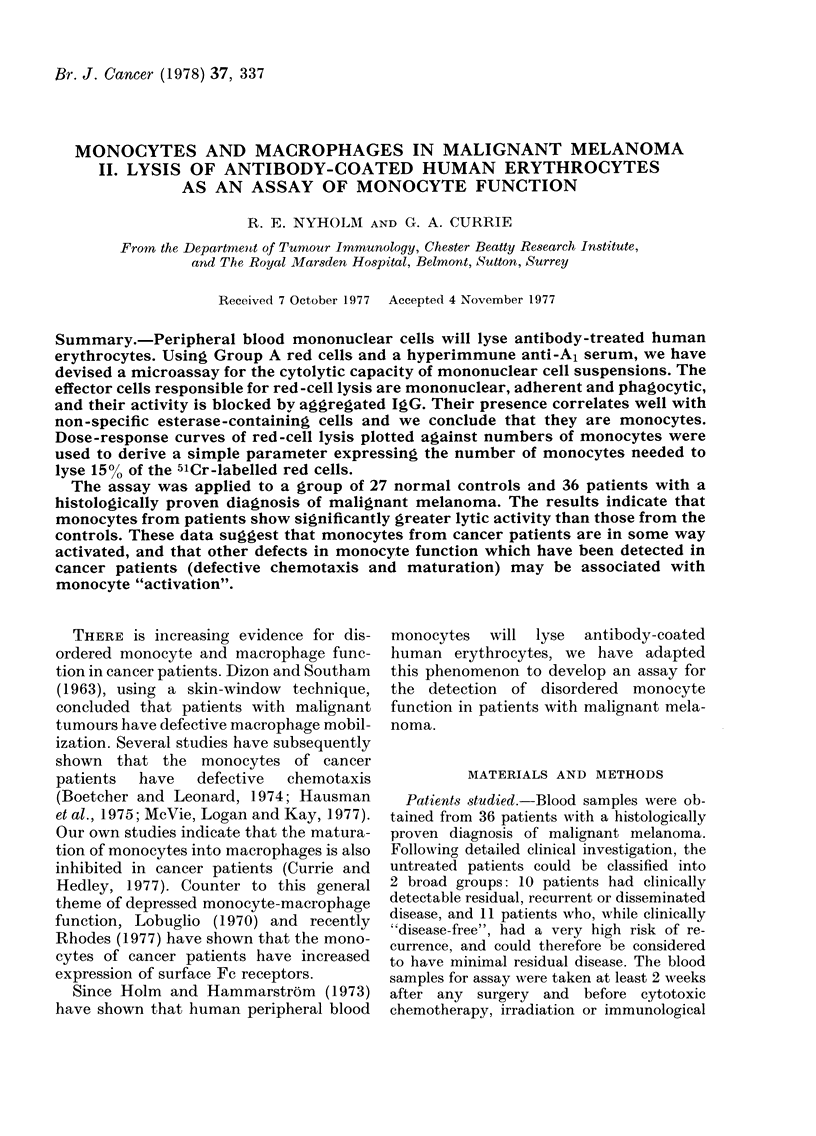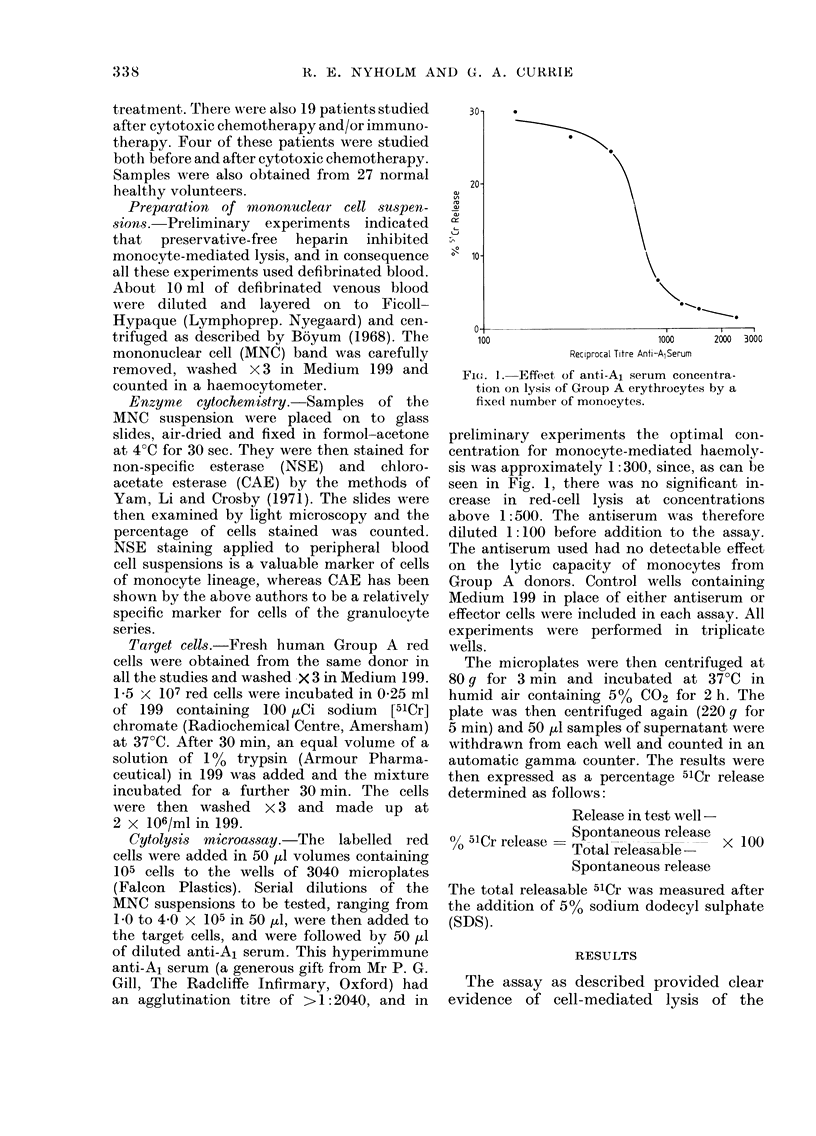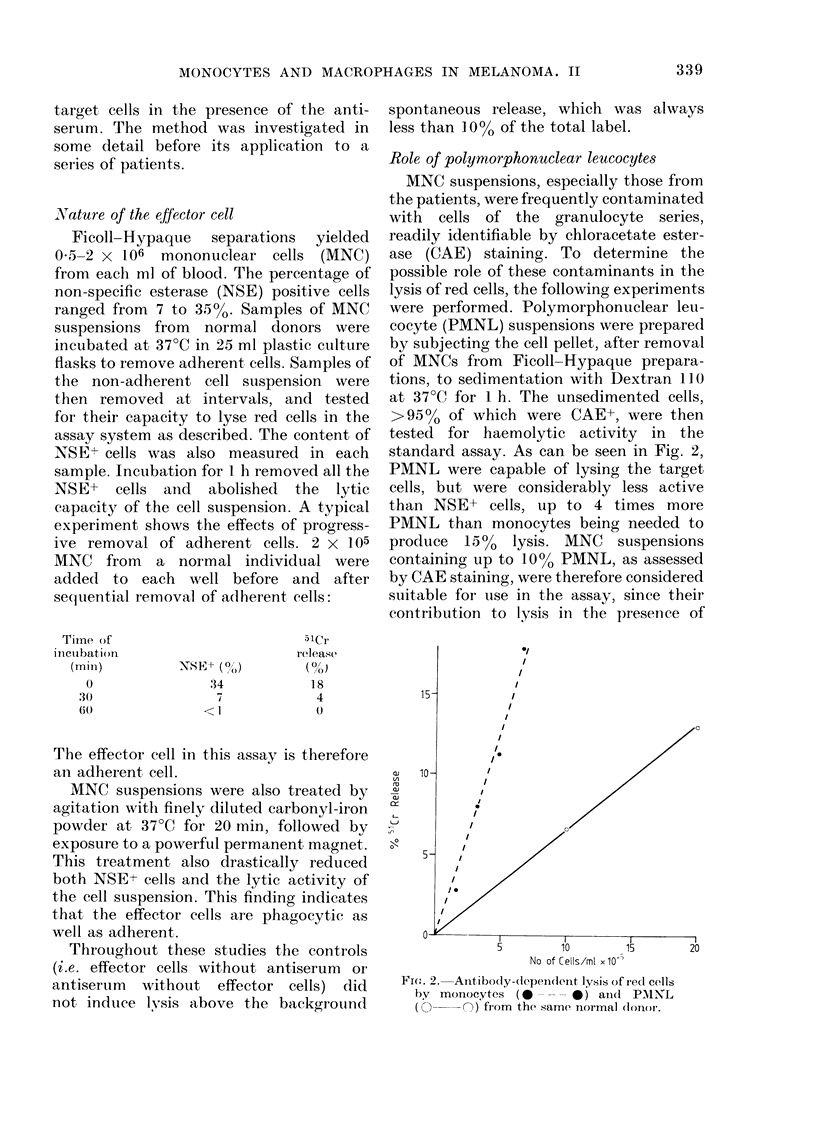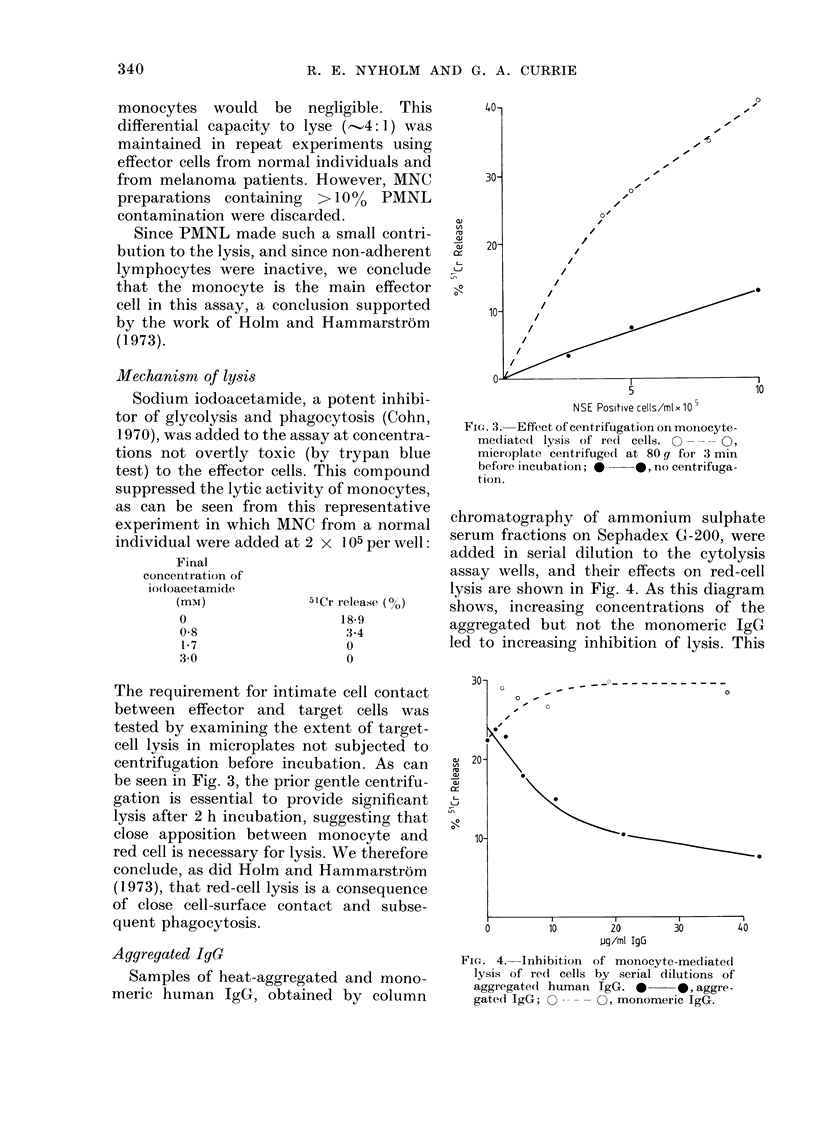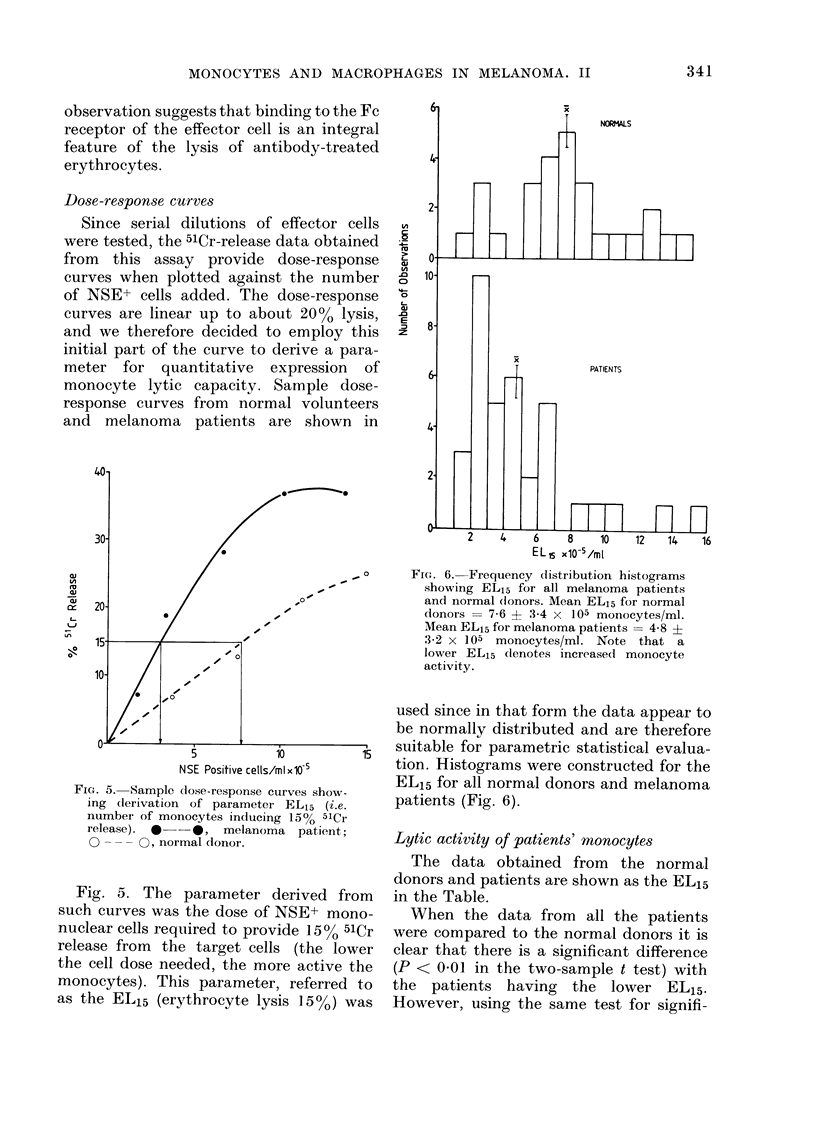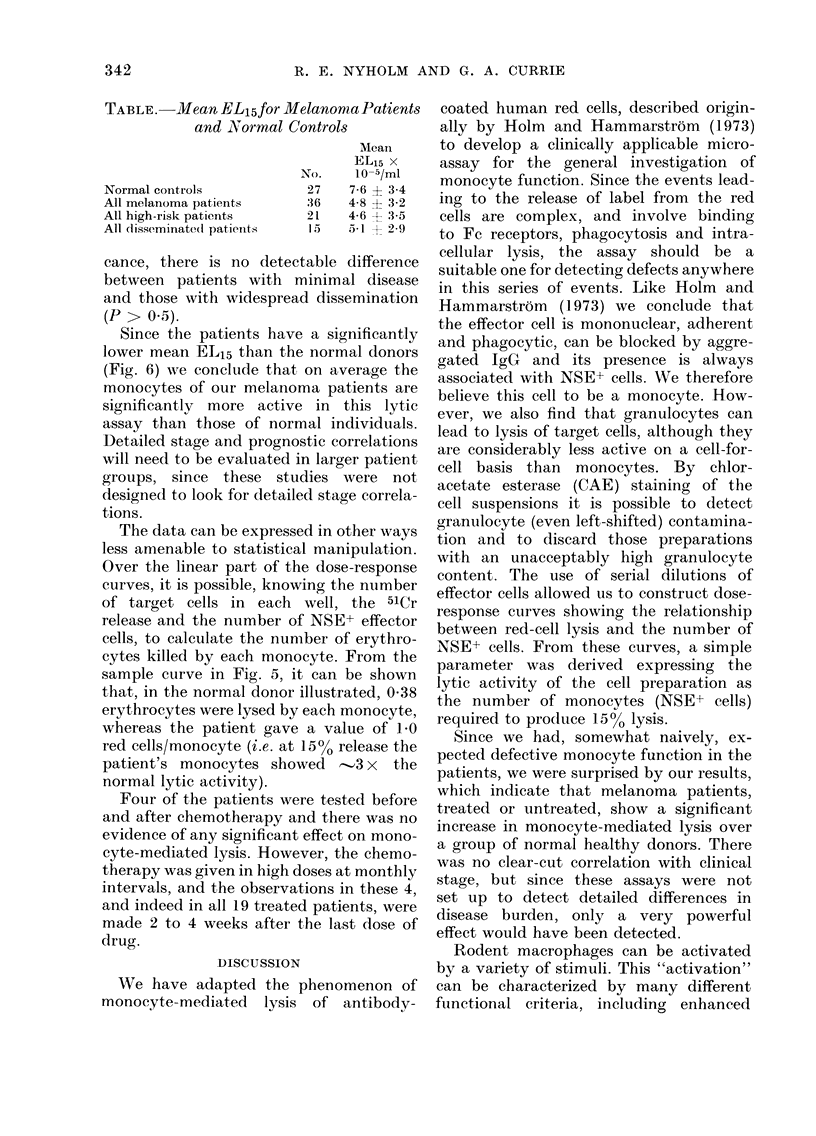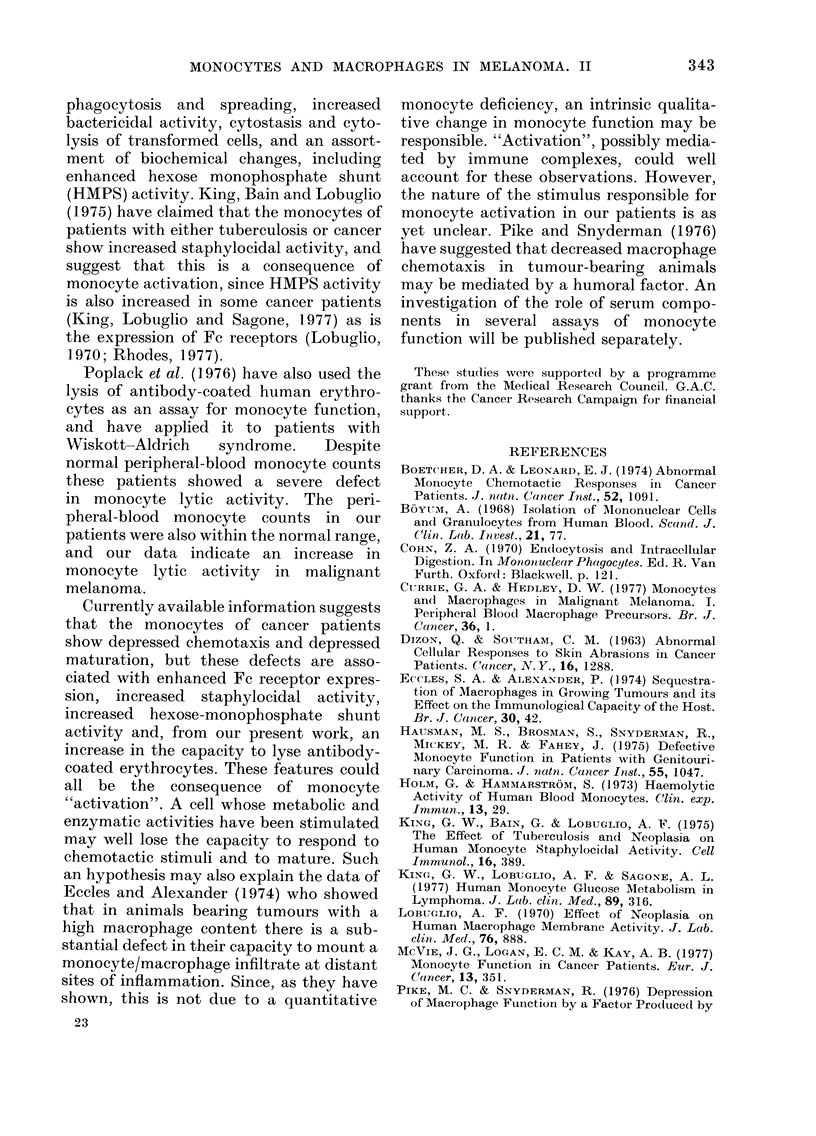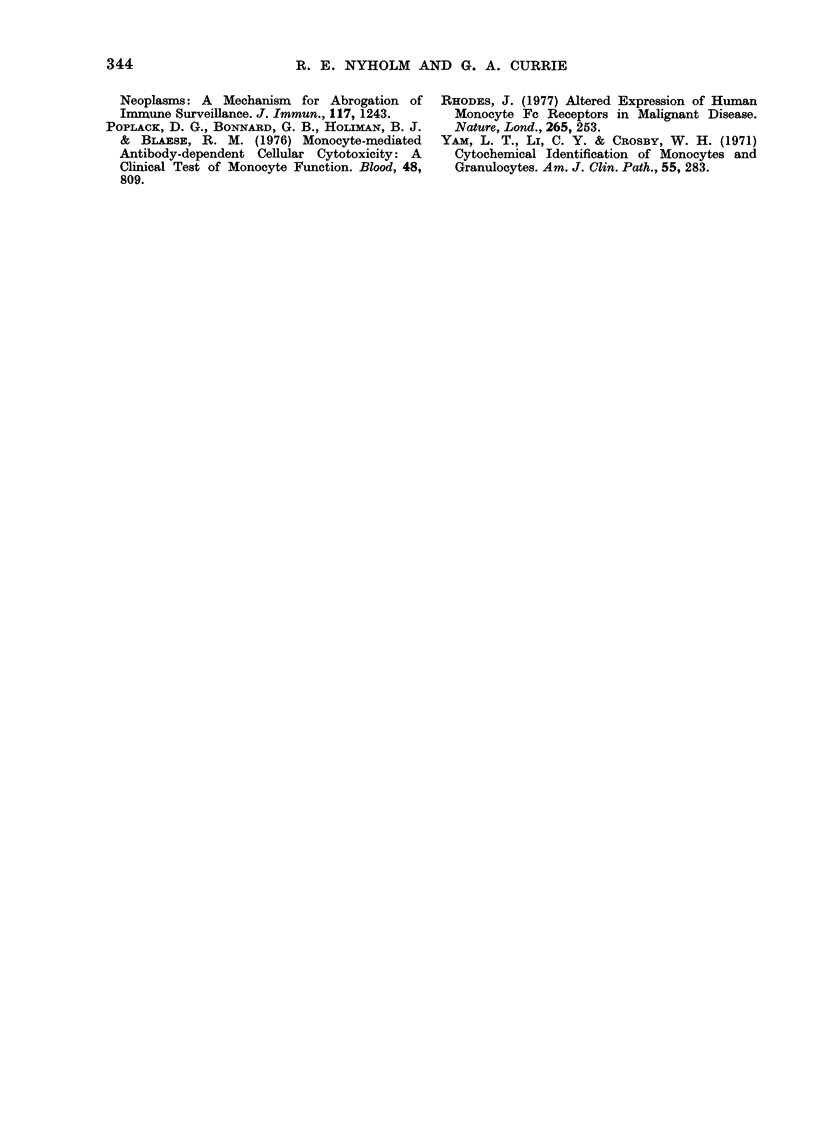# Monocytes and macrophages in malignant melanoma. II. Lysis of antibody-coated human erythrocytes as an assay of monocyte function.

**DOI:** 10.1038/bjc.1978.52

**Published:** 1978-03

**Authors:** R. E. Nyholm, G. A. Currie

## Abstract

Peripheral blood mononuclear cells will lyse antibody-treated human erythrocytes. Using Group A red cells and a hyperimmune anti-A1 serum, we have devised a microassay for the cytolytic capacity of mononuclear cell suspensions. The effector cells responsible for red-cell lysis are mononuclear, adherent and phagocytic, and their activity is blocked by aggregated IgG. Their presence correlates well with non-specific esterase-containing cells and we conclude that they are monocytes. Dose-response curves of red-cell lysis plotted against numbers of monocytes were used to derive a simple parameter expressing the number of monocytes needed to lyse 15% of the 51Cr-labelled red cells. The assay was applied to a group of 27 normal controls and 36 patients with a histologically proven diagnosis of malignant melanoma. The results indicate that monocytes from patients show significantly greater lytic activity than those from the controls. These data suggest that monocytes from cancer patients are in some way activated, and that other defects in monocyte function which have been detected in cancer patients (defective chemotaxis and maturation) may be associated with monocyte "activation".


*Br. J. Cancer* (1978) **37**, 337

### MONOCYTES AND MACROPHAGES IN MALIGNANT MELANOMA II. LYSIS OF ANTIBODY-COATED HUMAN ERYTHROCYTES AS AN ASSAY OF MONOCYTE FUNCTION

R. E. NYHOLM AND G. A. CURRIE

*From the Department of Tumour Immunology, Chester Beatty Research Institute, and The Royal Marsden Hospital, Belmont, Sutton, Surrey*

Received 7 October 1977 Accepted 4 November 1977

**Summary.—Peripheral blood mononuclear cells will lyse antibody-treated human erythrocytes. Using Group A red cells and a hyperimmune anti-$A_1$ serum, we have devised a microassay for the cytolytic capacity of mononuclear cell suspensions. The effector cells responsible for red-cell lysis are mononuclear, adherent and phagocytic, and their activity is blocked by aggregated IgG. Their presence correlates well with non-specific esterase-containing cells and we conclude that they are monocytes. Dose-response curves of red-cell lysis plotted against numbers of monocytes were used to derive a simple parameter expressing the number of monocytes needed to lyse 15% of the $^{51}$Cr-labelled red cells.**

**The assay was applied to a group of 27 normal controls and 36 patients with a histologically proven diagnosis of malignant melanoma. The results indicate that monocytes from patients show significantly greater lytic activity than those from the controls. These data suggest that monocytes from cancer patients are in some way activated, and that other defects in monocyte function which have been detected in cancer patients (defective chemotaxis and maturation) may be associated with monocyte "activation".**

THERE is increasing evidence for disordered monocyte and macrophage function in cancer patients. Dizon and Southam (1963), using a skin-window technique, concluded that patients with malignant tumours have defective macrophage mobilization. Several studies have subsequently shown that the monocytes of cancer patients have defective chemotaxis (Boetcher and Leonard, 1974; Hausman *et al.*, 1975; McVie, Logan and Kay, 1977). Our own studies indicate that the maturation of monocytes into macrophages is also inhibited in cancer patients (Currie and Hedley, 1977). Counter to this general theme of depressed monocyte-macrophage function, Lobuglio (1970) and recently Rhodes (1977) have shown that the monocytes of cancer patients have increased expression of surface Fc receptors.

Since Holm and Hammarström (1973) have shown that human peripheral blood monocytes will lyse antibody-coated human erythrocytes, we have adapted this phenomenon to develop an assay for the detection of disordered monocyte function in patients with malignant melanoma.

### MATERIALS AND METHODS

*Patients studied.*—Blood samples were obtained from 36 patients with a histologically proven diagnosis of malignant melanoma. Following detailed clinical investigation, the untreated patients could be classified into 2 broad groups: 10 patients had clinically detectable residual, recurrent or disseminated disease, and 11 patients who, while clinically "disease-free", had a very high risk of recurrence, and could therefore be considered to have minimal residual disease. The blood samples for assay were taken at least 2 weeks after any surgery and before cytotoxic chemotherapy, irradiation or immunological treatment. There were also 19 patients studied after cytotoxic chemotherapy and/or immunotherapy. Four of these patients were studied both before and after cytotoxic chemotherapy. Samples were also obtained from 27 normal healthy volunteers.

*Preparation of mononuclear cell suspensions.*—Preliminary experiments indicated that preservative-free heparin inhibited monocyte-mediated lysis, and in consequence all these experiments used defibrinated blood. About 10 ml of defibrinated venous blood were diluted and layered on to Ficoll–Hypaque (Lymphoprep. Nyegaard) and centrifuged as described by Böyum (1968). The mononuclear cell (MNC) band was carefully removed, washed ×3 in Medium 199 and counted in a haemocytometer.

*Enzyme cytochemistry.*—Samples of the MNC suspension were placed on to glass slides, air-dried and fixed in formol–acetone at 4°C for 30 sec. They were then stained for non-specific esterase (NSE) and chloroacetate esterase (CAE) by the methods of Yam, Li and Crosby (1971). The slides were then examined by light microscopy and the percentage of cells stained was counted. NSE staining applied to peripheral blood cell suspensions is a valuable marker of cells of monocyte lineage, whereas CAE has been shown by the above authors to be a relatively specific marker for cells of the granulocyte series.

*Target cells.*—Fresh human Group A red cells were obtained from the same donor in all the studies and washed ×3 in Medium 199. $1 \cdot 5 \times 10^7$ red cells were incubated in 0·25 ml of 199 containing 100 $\mu$Ci sodium [$^{51}$Cr] chromate (Radiochemical Centre, Amersham) at 37°C. After 30 min, an equal volume of a solution of 1% trypsin (Armour Pharmaceutical) in 199 was added and the mixture incubated for a further 30 min. The cells were then washed ×3 and made up at $2 \times 10^6$/ml in 199.

*Cytolysis microassay.*—The labelled red cells were added in 50 $\mu$l volumes containing $10^5$ cells to the wells of 3040 microplates (Falcon Plastics). Serial dilutions of the MNC suspensions to be tested, ranging from $1 \cdot 0$ to $4 \cdot 0 \times 10^5$ in 50 $\mu$l, were then added to the target cells, and were followed by 50 $\mu$l of diluted anti-$A_1$ serum. This hyperimmune anti-$A_1$ serum (a generous gift from Mr P. G. Gill, The Radcliffe Infirmary, Oxford) had an agglutination titre of >1:2040, and in

FIG. 1.—Effect of anti-$A_1$ serum concentration on lysis of Group A erythrocytes by a fixed number of monocytes.

preliminary experiments the optimal concentration for monocyte-mediated haemolysis was approximately 1:300, since, as can be seen in Fig. 1, there was no significant increase in red-cell lysis at concentrations above 1:500. The antiserum was therefore diluted 1:100 before addition to the assay. The antiserum used had no detectable effect on the lytic capacity of monocytes from Group A donors. Control wells containing Medium 199 in place of either antiserum or effector cells were included in each assay. All experiments were performed in triplicate wells.

The microplates were then centrifuged at 80 *g* for 3 min and incubated at 37°C in humid air containing 5% $CO_2$ for 2 h. The plate was then centrifuged again (220 *g* for 5 min) and 50 $\mu$l samples of supernatant were withdrawn from each well and counted in an automatic gamma counter. The results were then expressed as a percentage $^{51}$Cr release determined as follows:

$$\% \; ^{51}\text{Cr release} = \frac{\text{Release in test well} - \text{Spontaneous release}}{\text{Total releasable} - \text{Spontaneous release}} \times 100$$

The total releasable $^{51}$Cr was measured after the addition of 5% sodium dodecyl sulphate (SDS).

### RESULTS

The assay as described provided clear evidence of cell-mediated lysis of the target cells in the presence of the antiserum. The method was investigated in some detail before its application to a series of patients.

*Nature of the effector cell*

Ficoll–Hypaque separations yielded 0·5–2 × $10^6$ mononuclear cells (MNC) from each ml of blood. The percentage of non-specific esterase (NSE) positive cells ranged from 7 to 35%. Samples of MNC suspensions from normal donors were incubated at 37°C in 25 ml plastic culture flasks to remove adherent cells. Samples of the non-adherent cell suspension were then removed at intervals, and tested for their capacity to lyse red cells in the assay system as described. The content of $NSE^+$ cells was also measured in each sample. Incubation for 1 h removed all the $NSE^+$ cells and abolished the lytic capacity of the cell suspension. A typical experiment shows the effects of progressive removal of adherent cells. 2 × $10^5$ MNC from a normal individual were added to each well before and after sequential removal of adherent cells:

| Time of incubation (min) | $NSE^+$ (%) | $^{51}Cr$ release (%) |
|---|---|---|
| 0 | 34 | 18 |
| 30 | 7 | 4 |
| 60 | <1 | 0 |

The effector cell in this assay is therefore an adherent cell.

MNC suspensions were also treated by agitation with finely diluted carbonyl-iron powder at 37°C for 20 min, followed by exposure to a powerful permanent magnet. This treatment also drastically reduced both $NSE^+$ cells and the lytic activity of the cell suspension. This finding indicates that the effector cells are phagocytic as well as adherent.

Throughout these studies the controls (*i.e.* effector cells without antiserum or antiserum without effector cells) did not induce lysis above the background spontaneous release, which was always less than 10% of the total label.

*Role of polymorphonuclear leucocytes*

MNC suspensions, especially those from the patients, were frequently contaminated with cells of the granulocyte series, readily identifiable by chloracetate esterase (CAE) staining. To determine the possible role of these contaminants in the lysis of red cells, the following experiments were performed. Polymorphonuclear leucocyte (PMNL) suspensions were prepared by subjecting the cell pellet, after removal of MNCs from Ficoll–Hypaque preparations, to sedimentation with Dextran 110 at 37°C for 1 h. The unsedimented cells, >95% of which were $CAE^+$, were then tested for haemolytic activity in the standard assay. As can be seen in Fig. 2, PMNL were capable of lysing the target cells, but were considerably less active than $NSE^+$ cells, up to 4 times more PMNL than monocytes being needed to produce 15% lysis. MNC suspensions containing up to 10% PMNL, as assessed by CAE staining, were therefore considered suitable for use in the assay, since their contribution to lysis in the presence of

FIG. 2.—Antibody-dependent lysis of red cells by monocytes (● – – – ●) and PMNL (○——○) from the same normal donor.

monocytes would be negligible. This differential capacity to lyse (~4:1) was maintained in repeat experiments using effector cells from normal individuals and from melanoma patients. However, MNC preparations containing >10% PMNL contamination were discarded.

Since PMNL made such a small contribution to the lysis, and since non-adherent lymphocytes were inactive, we conclude that the monocyte is the main effector cell in this assay, a conclusion supported by the work of Holm and Hammarström (1973).

*Mechanism of lysis*

Sodium iodoacetamide, a potent inhibitor of glycolysis and phagocytosis (Cohn, 1970), was added to the assay at concentrations not overtly toxic (by trypan blue test) to the effector cells. This compound suppressed the lytic activity of monocytes, as can be seen from this representative experiment in which MNC from a normal individual were added at 2 × $10^5$ per well:

| Final concentration of iodoacetamide (mM) | $^{51}$Cr release (%) |
|---|---|
| 0 | 18·9 |
| 0·8 | 3·4 |
| 1·7 | 0 |
| 3·0 | 0 |

The requirement for intimate cell contact between effector and target cells was tested by examining the extent of target-cell lysis in microplates not subjected to centrifugation before incubation. As can be seen in Fig. 3, the prior gentle centrifugation is essential to provide significant lysis after 2 h incubation, suggesting that close apposition between monocyte and red cell is necessary for lysis. We therefore conclude, as did Holm and Hammarström (1973), that red-cell lysis is a consequence of close cell-surface contact and subsequent phagocytosis.

*Aggregated IgG*

Samples of heat-aggregated and monomeric human IgG, obtained by column

FIG. 3.—Effect of centrifugation on monocyte-mediated lysis of red cells. ○ – – – ○, microplate centrifuged at 80 *g* for 3 min before incubation; ●——●, no centrifugation.

chromatography of ammonium sulphate serum fractions on Sephadex G-200, were added in serial dilution to the cytolysis assay wells, and their effects on red-cell lysis are shown in Fig. 4. As this diagram shows, increasing concentrations of the aggregated but not the monomeric IgG led to increasing inhibition of lysis. This

FIG. 4.—Inhibition of monocyte-mediated lysis of red cells by serial dilutions of aggregated human IgG. ●——●, aggregated IgG; ○ – – – ○, monomeric IgG.

observation suggests that binding to the Fc receptor of the effector cell is an integral feature of the lysis of antibody-treated erythrocytes.

*Dose-response curves*

Since serial dilutions of effector cells were tested, the $^{51}Cr$-release data obtained from this assay provide dose-response curves when plotted against the number of $NSE^+$ cells added. The dose-response curves are linear up to about 20% lysis, and we therefore decided to employ this initial part of the curve to derive a parameter for quantitative expression of monocyte lytic capacity. Sample dose-response curves from normal volunteers and melanoma patients are shown in

FIG. 5.—Sample dose-response curves showing derivation of parameter $EL_{15}$ (*i.e.* number of monocytes inducing 15% $^{51}Cr$ release). ●——●, melanoma patient; ○ - - - ○, normal donor.

Fig. 5. The parameter derived from such curves was the dose of $NSE^+$ mononuclear cells required to provide 15% $^{51}Cr$ release from the target cells (the lower the cell dose needed, the more active the monocytes). This parameter, referred to as the $EL_{15}$ (erythrocyte lysis 15%) was

FIG. 6.—Frequency distribution histograms showing $EL_{15}$ for all melanoma patients and normal donors. Mean $EL_{15}$ for normal donors $= 7 \cdot 6 \pm 3 \cdot 4 \times 10^5$ monocytes/ml. Mean $EL_{15}$ for melanoma patients $= 4 \cdot 8 \pm 3 \cdot 2 \times 10^5$ monocytes/ml. Note that a lower $EL_{15}$ denotes increased monocyte activity.

used since in that form the data appear to be normally distributed and are therefore suitable for parametric statistical evaluation. Histograms were constructed for the $EL_{15}$ for all normal donors and melanoma patients (Fig. 6).

*Lytic activity of patients' monocytes*

The data obtained from the normal donors and patients are shown as the $EL_{15}$ in the Table.

When the data from all the patients were compared to the normal donors it is clear that there is a significant difference ($P < 0 \cdot 01$ in the two-sample $t$ test) with the patients having the lower $EL_{15}$. However, using the same test for signifi-

TABLE.—*Mean $EL_{15}$ for Melanoma Patients and Normal Controls*

| | No. | Mean $EL_{15}\times 10^{-5}$/ml |
|---|---|---|
| Normal controls | 27 | $7\cdot6 \pm 3\cdot4$ |
| All melanoma patients | 36 | $4\cdot8 \pm 3\cdot2$ |
| All high-risk patients | 21 | $4\cdot6 \pm 3\cdot5$ |
| All disseminated patients | 15 | $5\cdot1 \pm 2\cdot9$ |

cance, there is no detectable difference between patients with minimal disease and those with widespread dissemination ($P > 0\cdot5$).

Since the patients have a significantly lower mean $EL_{15}$ than the normal donors (Fig. 6) we conclude that on average the monocytes of our melanoma patients are significantly more active in this lytic assay than those of normal individuals. Detailed stage and prognostic correlations will need to be evaluated in larger patient groups, since these studies were not designed to look for detailed stage correlations.

The data can be expressed in other ways less amenable to statistical manipulation. Over the linear part of the dose-response curves, it is possible, knowing the number of target cells in each well, the $^{51}Cr$ release and the number of $NSE^+$ effector cells, to calculate the number of erythrocytes killed by each monocyte. From the sample curve in Fig. 5, it can be shown that, in the normal donor illustrated, 0·38 erythrocytes were lysed by each monocyte, whereas the patient gave a value of 1·0 red cells/monocyte (*i.e.* at 15% release the patient's monocytes showed $\sim 3\times$ the normal lytic activity).

Four of the patients were tested before and after chemotherapy and there was no evidence of any significant effect on monocyte-mediated lysis. However, the chemotherapy was given in high doses at monthly intervals, and the observations in these 4, and indeed in all 19 treated patients, were made 2 to 4 weeks after the last dose of drug.

### DISCUSSION

We have adapted the phenomenon of monocyte-mediated lysis of antibody-coated human red cells, described originally by Holm and Hammarström (1973) to develop a clinically applicable microassay for the general investigation of monocyte function. Since the events leading to the release of label from the red cells are complex, and involve binding to Fc receptors, phagocytosis and intracellular lysis, the assay should be a suitable one for detecting defects anywhere in this series of events. Like Holm and Hammarström (1973) we conclude that the effector cell is mononuclear, adherent and phagocytic, can be blocked by aggregated IgG and its presence is always associated with $NSE^+$ cells. We therefore believe this cell to be a monocyte. However, we also find that granulocytes can lead to lysis of target cells, although they are considerably less active on a cell-for-cell basis than monocytes. By chloracetate esterase (CAE) staining of the cell suspensions it is possible to detect granulocyte (even left-shifted) contamination and to discard those preparations with an unacceptably high granulocyte content. The use of serial dilutions of effector cells allowed us to construct dose-response curves showing the relationship between red-cell lysis and the number of $NSE^+$ cells. From these curves, a simple parameter was derived expressing the lytic activity of the cell preparation as the number of monocytes ($NSE^+$ cells) required to produce 15% lysis.

Since we had, somewhat naively, expected defective monocyte function in the patients, we were surprised by our results, which indicate that melanoma patients, treated or untreated, show a significant increase in monocyte-mediated lysis over a group of normal healthy donors. There was no clear-cut correlation with clinical stage, but since these assays were not set up to detect detailed differences in disease burden, only a very powerful effect would have been detected.

Rodent macrophages can be activated by a variety of stimuli. This "activation" can be characterized by many different functional criteria, including enhanced phagocytosis and spreading, increased bactericidal activity, cytostasis and cytolysis of transformed cells, and an assortment of biochemical changes, including enhanced hexose monophosphate shunt (HMPS) activity. King, Bain and Lobuglio (1975) have claimed that the monocytes of patients with either tuberculosis or cancer show increased staphylocidal activity, and suggest that this is a consequence of monocyte activation, since HMPS activity is also increased in some cancer patients (King, Lobuglio and Sagone, 1977) as is the expression of Fc receptors (Lobuglio, 1970; Rhodes, 1977).

Poplack *et al.* (1976) have also used the lysis of antibody-coated human erythrocytes as an assay for monocyte function, and have applied it to patients with Wiskott–Aldrich syndrome. Despite normal peripheral-blood monocyte counts these patients showed a severe defect in monocyte lytic activity. The peripheral-blood monocyte counts in our patients were also within the normal range, and our data indicate an increase in monocyte lytic activity in malignant melanoma.

Currently available information suggests that the monocytes of cancer patients show depressed chemotaxis and depressed maturation, but these defects are associated with enhanced Fc receptor expression, increased staphylocidal activity, increased hexose-monophosphate shunt activity and, from our present work, an increase in the capacity to lyse antibody-coated erythrocytes. These features could all be the consequence of monocyte "activation". A cell whose metabolic and enzymatic activities have been stimulated may well lose the capacity to respond to chemotactic stimuli and to mature. Such an hypothesis may also explain the data of Eccles and Alexander (1974) who showed that in animals bearing tumours with a high macrophage content there is a substantial defect in their capacity to mount a monocyte/macrophage infiltrate at distant sites of inflammation. Since, as they have shown, this is not due to a quantitative monocyte deficiency, an intrinsic qualitative change in monocyte function may be responsible. "Activation", possibly mediated by immune complexes, could well account for these observations. However, the nature of the stimulus responsible for monocyte activation in our patients is as yet unclear. Pike and Snyderman (1976) have suggested that decreased macrophage chemotaxis in tumour-bearing animals may be mediated by a humoral factor. An investigation of the role of serum components in several assays of monocyte function will be published separately.

These studies were supported by a programme grant from the Medical Research Council. G.A.C. thanks the Cancer Research Campaign for financial support.

REFERENCES

BOETCHER, D. A. & LEONARD, E. J. (1974) Abnormal Monocyte Chemotactic Responses in Cancer Patients. *J. natn. Cancer Inst.*, **52**, 1091.

BÖYUM, A. (1968) Isolation of Mononuclear Cells and Granulocytes from Human Blood. *Scand. J. Clin. Lab. Invest.*, **21**, 77.

COHN, Z. A. (1970) Endocytosis and Intracellular Digestion. In *Mononuclear Phagocytes*. Ed. R. Van Furth. Oxford: Blackwell. p. 121.

CURRIE, G. A. & HEDLEY, D. W. (1977) Monocytes and Macrophages in Malignant Melanoma. I. Peripheral Blood Macrophage Precursors. *Br. J. Cancer*, **36**, 1.

DIZON, Q. & SOUTHAM, C. M. (1963) Abnormal Cellular Responses to Skin Abrasions in Cancer Patients. *Cancer, N.Y.*, **16**, 1288.

ECCLES, S. A. & ALEXANDER, P. (1974) Sequestration of Macrophages in Growing Tumours and its Effect on the Immunological Capacity of the Host. *Br. J. Cancer*, **30**, 42.

HAUSMAN, M. S., BROSMAN, S., SNYDERMAN, R., MICKEY, M. R. & FAHEY, J. (1975) Defective Monocyte Function in Patients with Genitourinary Carcinoma. *J. natn. Cancer Inst.*, **55**, 1047.

HOLM, G. & HAMMARSTRÖM, S. (1973) Haemolytic Activity of Human Blood Monocytes. *Clin. exp. Immun.*, **13**, 29.

KING, G. W., BAIN, G. & LOBUGLIO, A. F. (1975) The Effect of Tuberculosis and Neoplasia on Human Monocyte Staphylocidal Activity. *Cell Immunol.*, **16**, 389.

KING, G. W., LOBUGLIO, A. F. & SAGONE, A. L. (1977) Human Monocyte Glucose Metabolism in Lymphoma. *J. Lab. clin. Med.*, **89**, 316.

LOBUGLIO, A. F. (1970) Effect of Neoplasia on Human Macrophage Membrane Activity. *J. Lab. clin. Med.*, **76**, 888.

MCVIE, J. G., LOGAN, E. C. M. & KAY, A. B. (1977) Monocyte Function in Cancer Patients. *Eur. J. Cancer*, **13**, 351.

PIKE, M. C. & SNYDERMAN, R. (1976) Depression of Macrophage Function by a Factor Produced by

Neoplasms: A Mechanism for Abrogation of Immune Surveillance. *J. Immun.*, **117**, 1243.

POPLACK, D. G., BONNARD, G. B., HOLIMAN, B. J. & BLAESE, R. M. (1976) Monocyte-mediated Antibody-dependent Cellular Cytotoxicity: A Clinical Test of Monocyte Function. *Blood*, **48**, 809.

RHODES, J. (1977) Altered Expression of Human Monocyte Fc Receptors in Malignant Disease. *Nature, Lond.*, **265**, 253.

YAM, L. T., LI, C. Y. & CROSBY, W. H. (1971) Cytochemical Identification of Monocytes and Granulocytes. *Am. J. Clin. Path.*, **55**, 283.